# Cloning retinoid and peroxisome proliferator-activated nuclear receptors of the Pacific oyster and *in silico* binding to environmental chemicals

**DOI:** 10.1371/journal.pone.0176024

**Published:** 2017-04-20

**Authors:** Susanne Vogeler, Tamara S. Galloway, Michail Isupov, Tim P. Bean

**Affiliations:** 1 School of Biosciences, College of Life and Environmental Sciences, University of Exeter, Exeter, United Kingdom; 2 Centre for Environment, Fisheries and Aquaculture Science, Cefas Weymouth Laboratory, Weymouth, United Kingdom; Laboratoire de Biologie du Développement de Villefranche-sur-Mer, FRANCE

## Abstract

Disruption of nuclear receptors, a transcription factor superfamily regulating gene expression in animals, is one proposed mechanism through which pollution causes effects in aquatic invertebrates. Environmental pollutants have the ability to interfere with the receptor’s functions through direct binding and inducing incorrect signals. Limited knowledge of invertebrate endocrinology and molecular regulatory mechanisms, however, impede the understanding of endocrine disruptive effects in many aquatic invertebrate species. Here, we isolated three nuclear receptors of the Pacific oyster, *Crassostrea gigas*: two isoforms of the retinoid X receptor, CgRXR-1 and CgRXR-2, a retinoic acid receptor ortholog CgRAR, and a peroxisome proliferator-activated receptor ortholog CgPPAR. Computer modelling of the receptors based on 3D crystal structures of human proteins was used to predict each receptor’s ability to bind to different ligands *in silico*. CgRXR showed high potential to bind and be activated by 9-*cis* retinoic acid and the organotin tributyltin (TBT). Computer modelling of CgRAR revealed six residues in the ligand binding domain, which prevent the successful interaction with natural and synthetic retinoid ligands. This supports an existing theory of loss of retinoid binding in molluscan RARs. Modelling of CgPPAR was less reliable due to high discrepancies in sequence to its human ortholog. Yet, there are suggestions of binding to TBT, but not to rosiglitazone. The effect of potential receptor ligands on early oyster development was assessed after 24h of chemical exposure. TBT oxide (0.2μg/l), all-*trans* retinoic acid (ATRA) (0.06 mg/L) and perfluorooctanoic acid (20 mg/L) showed high effects on development (>74% abnormal developed D-shelled larvae), while rosiglitazone (40 mg/L) showed no effect. The results are discussed in relation to a putative direct (TBT) disruption effect on nuclear receptors. The inability of direct binding of ATRA to CgRAR suggests either a disruptive effect through a pathway excluding nuclear receptors or an indirect interaction. Our findings provide valuable information on potential mechanisms of molluscan nuclear receptors and the effects of environmental pollution on aquatic invertebrates.

## Introduction

One specific mechanism through which endocrine disrupting pollutants can affect wildlife is the disruption of gene expression regulation by interfering with the function of nuclear receptors. Nuclear receptors (NR) are ligand binding transcription factors in metazoan species, regulating the transcription of many fundamental genes involved in development, reproduction and homeostasis. These receptors bind to specific response elements in a gene promotor sequence [1] and function either as monomers, homodimers, or heterodimers [2]. A subset of these receptors are able to interact with ligands including endogenous or exogenous organic compounds, such as steroids, thyroid hormones and retinoids, which operate either as agonist or antagonist [1]. Environmental pollutants can have the same ability to interact with NRs and subsequently induce incorrect signalling. Xenobiotic agonists of NRs are commonly cited as a key mode of action in events of endocrine disruption [3, 4].

A classic example for aquatic pollution, which is hypothesised to be linked to NR disruption, is the effect of tributyltin (TBT) on coastal wildlife. This synthetic organotin was introduced as an effective active ingredient of antifouling paints in the 1960s and banned world-wide in 2008 [5, 6]. The leaching and accumulation of TBT into the environment resulted in severe effects on marine wildlife. In gastropods, exposure to TBT causes female masculinisation including imposex, a superimposition of male genitalia on female gastropods [7]. In bivalves, such as the Pacific oyster, *Crassostrea gigas*, shell thickening, growth reduction, developmental disruption and a high rate of mortality were observed [8–13]. It is currently assumed that TBT alters the normal function of a specific NR, the retinoid X receptor RXR [14, 15], and reports on TBT as a ligand for RXR orthologs in a snail gastropod species [16] and human [17, 18] support this theory.

Disruption of RXR by pollutants like TBT could also affect other NRs. The RXR receptor is the predominant heterodimer partner for NRs in various species [19]. Heterodimer constructs can be activated through a ligand by binding to both partners (permissive) or just to the RXR partner (non-permissive) [20]. The peroxisome proliferator-activated receptor (PPARs) is a common permissive partner of RXRs in vertebrates [21]. The RXR/PPAR heterodimer has been proposed as the pathway, through which TBT has its disruptive effect by binding to the RXR and then possibly intensifying the effect by also binding to PPAR [17, 18, 22, 23]. In molluscan species PPAR homologs have been identified, although not characterised [24, 25].

The RXR belong to the retinoid activated receptors and 9-*cis* retinoic acid (9RA), a vitamin A derivate, is the proposed natural ligand [26, 27]. In vertebrates, retinoic acids (RAs) are morphogens involved in pattern formation, cell differentiation and proliferation as well as embryonic development and reproduction [28–30]. 9RA and other RA derivatives such as all*-trans* RA (ATRA) and 13-*cis* RA are also natural ligands for another retinoic activated receptor type RAR (retinoic acid receptor) [31]. RAR is another RXR partner (although non-permissive) [32, 33]. However, RAs are among the most potent known teratogens for animals [34]. When present at an inappropriate titre or time point, RAs affect normal development via binding to retinoid receptors (RXR and RAR) and initialising incorrect signals [35]. Many xenobiotic pollutants like organochlorine pesticides, styrene dimers, monoalkylphenol and parabens have been identified as RAR agonists [36]. Disruptive RAR agonistic activity by unidentified pollutants has also been detected in aquatic environments in North America and Asia [37]. Research on several gastropod species has shown that exposure to natural and synthetic RAs causes eye defects, shell malformation, incorrect neuronal differentiation and abnormalities in sex organ development [38–40]. RAR orthologs have previously been characterised in only a few gastropod species [39, 41, 42], but all of them seem to be unresponsive to RAs [41, 42]. However, research on the RA machinery including RARs in invertebrates is limited and includes only three gastropod species and no other molluscan or protostome representatives.

Molluscs are the second largest group of invertebrates and represent roughly a quarter of all characterised marine species. The presence of NRs has been established in many gastropods and bivalves [24, 25], but the exact functions of most receptors have yet to be discovered. Here we clone and characterise three NRs of the Pacific oyster, *C*. *gigas*: CgRXR, CgRAR and CgPPAR. All three receptors are phylogenetically assessed and their sequences are structurally analysed for typical features of NRs including the highly-conserved DNA binding domain (DBD) and the ligand binding domain (LBD), wherewith some receptors bind ligands. Additional *in silico* 3D modelling and computational docking are used to predict whether these receptors have the potential to bind to common natural and/or synthetic ligands of vertebrate and gastropod homologous receptors: 9RA, the natural ligand of RXR and RAR [26, 27, 31]; ATRA, another natural ligand of RAR [31]; arotinoid acid (TTNPB), a synthetic RAR agonist [43]; bis(tributyltin) oxide (TBTO), a synthetic ligand of RXR and human PPAR [16, 18, 22, 23]; rosiglitazone, a diabetic drug binding to human PPARγ receptors [44]; and perfluooctanoic acid (PFOA), a highly persistent synthetic compound, which interacts with vertebrate PPARα [45]. Exposure experiments are conducted using the hypothetical ligands (except 9RA) to test for potential effects on early oyster development.

## Material and methods

### Animals and chemical reagents

Male and female conditioned adult Pacific oyster (*C*. *gigas*) individuals were obtained from the Guernsey Sea Farm (Guernsey, UK). Individuals for RNA extraction were frozen in liquid nitrogen and stored at -80°C. Individual brood stocks were held in filtered (0.2 μm) and UV treated natural seawater (26.0 ‰; pH 7.8) at 10–15°C.

Bis(tributyltin) oxide (TBTO), rosiglitazone (Rosi), perfluooctanoic acid (PFOA), and all-*trans* retinoic acid (ATRA) were purchased from Sigma-Aldrich. Stock solution and dilutions were freshly prepared with dimethylsulfoxide (DMSO).

### Cloning *C*. *gigas* nuclear receptors CgRXR, CgRAR and CgPPAR

Oligonucleotide primers for sequencing the full length of the oyster NR sequences CgRXR, CgRAR and CgPPAR (S1 Table) were designed with Primer-Blast at NCBI [46] based on *C*. *gigas* genomic DNA data [47] for each gene (GenBank: CGI_10004075 (CgRXR); CGI_10028545 (CgRAR); CGI_10011509 (CgPPAR)). Total RNA was extracted from frozen whole adults and mixed embryo oyster individuals (embryo toxicity test). Extraction, DNA digestion, reverse transcription, and amplicon visualization and purification were performed as described previously [24]. Amplicons were obtained by RT-PCR under the following conditions: 95°C for 2 min, thirty cycles of 95°C for 30 s, 60°C for 30 s, 72°C for 2 min, and a final extension at 72°C for 5 min, and cloned into a pGEM-T Easy vector (Promega). Vectors were purified using the PureLink Plasmid miniprep kit (Invitrogen), and were subsequently sequenced by Eurofins MWG Operon (Cologne, Germany). Each identified receptor sequence was confirmed by three independent successful cloning attempts.

The obtained coding DNA sequences (CDS) for all receptors (GenBank accession numbers: CgRXR1: KX590999; CgRXR2: KX591000; CgRAR: KX591001; CgPPAR: KX591002) were aligned to their associated genomic DNA sequence to identify isoforms and their intron/exon structure.

### Protein analysis and phylogeny

The deduced amino acid sequences of each receptor (See section 2.2 for GenBank accession numbers. Protein sequences: S1 File), including different isoforms, were annotated by using the Conserved Domain Database at NCBI [48]. The sequence identities of each receptor domain were assessed against homologs of other closely related molluscan species (RXR: *Reishia clavigera*, *Nucella lapillus*, *Chlamys farreri*, *Biomphalaria glabrata*, *Lymnaea stagnalis;* RAR: *R*. *clavigera*, *N*. *lapillus*, *L*. *stagnalis;* PPAR: *B*. *glabrata*, *Lottia gigantea*). Identities to *Homo sapiens* receptors were also calculated.

Phylogeny of oyster receptors was inferred using the Maximum Likelihood and Bayesian Inference methods as previously described [24]. The DBD and selected parts of the LBD of each receptor were combined and then aligned with NR homologs of species across phyla (S2 File) using default parameters in MUSCLE v3.8.31 [49]. Maximum Likelihood phylogenetic tree was constructed using PhyML v3.0 [50] with an LG matrix plus optimized invariable sites (+I) and gamma distributed rate heterogeneity among sites (+G) and 1000 bootstrap replicates. The Bayesian Inference tree was calculated using MrBayes v3.2.2 [51] with the JTT+I+G model. Four randomly started simultaneous Markov chains were running for 5 million generations with chains sampled every 100 generations and a burnin of 5000 trees. The phylogenetic trees were visualized and illustrated with FigTree v1.4.0 (http://tree.bio.ed.ac.uk/software/figtree/).

### Three-dimensional (3D) modelling of CgRXR, CgRAR and CgPPAR LBD

Crystal structures of human RXR, RAR or PPAR LBD in complex with either 9RA (1FBY [52], 3LBD [53]), ATRA (2LBD [53]), TTNPB (1XAP [54]), TBT (3E94 [18]) or rosiglitazone (4EMA [55]) were obtained from the RCSB Protein Data Bank (PDB) [56]. Ligand dictionaries for docking were also obtained from the PDB site including the molecular structure of estradiol for negative control. *C*. *gigas*, *R*. *clavigera* and *N*. *lapillus* models for RXR and RAR and oyster models for PPAR were constructed by the modelling server SWISS-MODEL [57] using the crystal structures of the human NR LBDs with the investigated ligand bound as templates. Computational docking of ligands to human and generated mollusc NR models was conducted using AutoDock Vina [58] with AutoDockTools and Phython Molecular Viewer PMV graphical interface[59]. The mean ligand binding energy was estimated from three independent computational docking calculations with the assumption that more negative values equates stronger ligand binding. The tin atom was replaced with zinc for the docking calculation of TBT, since atom parameters for tin are not included in the AutoDock Vina dictionary [58]. Zinc was chosen as the best tin mimic among other metals available in the dictionary due to its similar tetragonal coordination and related preference to sulfhydryl side chains of cysteine residues. The programmes Coot [60] and CCP4 Molecular Graphics [61] were used for assessment and visualizing of ligand docking results.

### Embryo toxicity test

The embryo toxicity assays were executed following the standardised ICES oyster embryo bioassay (OEB) protocol [62]. Fertilisation was carried out in natural seawater using eggs from two females and a sperm mix of three male conditioned adult oyster individuals. Three hours post fertilisation (hpf) approximately 200 embryos/ml in a total volume of 250 ml were exposed to chemicals with two final concentrations (low & high): TBT low: 0.2 μg/L; TBT high: 2 μg/L; Rosi low: 4 μg/L; Rosi high: 40 μg/L; ATRA low: 0.06 mg/L; ATRA high: 0.6 mg/L; PFOA low: 20 mg/L; PFOA high: 50 mg/L. Additionally, water and DMSO (0.4%) controls were prepared and three replicates per treatment were performed. The three replicates are determined as pseudo-replicates, since the experiment took place on the same day and the same parental individuals, chemicals stocks and water source were used. As recommended by the OEB protocol, a gradient of zinc concentration was used as the reference toxicant. The assay was terminated 24 hours post fertilisation (hpf). Each pseudo-replicate treatment was well mixed and approximately 100 ml of trochophore larval stage (12 hpf) and approximately 150 ml of D-shelled larval stage (24 hpf) were concentrated to a smaller volume (5 ml) by retaining oyster individuals on a 20 μm filter. One millilitre of the concentrated samples was snap frozen and stored at -80°C for gene expression analysis. The remaining D-shelled larvae were preserved in a buffered formaldehyde solution (final concentration/sample 0.4% formaldehyde) for later observation. Per replicate, 100–140 oyster individuals were microscopically assessed based on their larval appearance and the numbers of perfect and abnormal D-shaped larvae were counted. Differential interference contrast microscopic pictures of exposed larvae were taken using a Nikon Eclipse E800 microscope and the Nikon element BR image analysis software. Abnormal larvae were further categorized: extruding velum, protruding soft tissue, partly developed shell, arrested growth/ shell not developed. Mean percentages including the standard error (±SE) for each larval category of each pseudo-replicate set were calculated. The rate of swimming larvae was calculated to investigate differences between treatments of normal larval development. Prior sampling for microscopic assessment D-shaped larval samples from the water column of each replicate were taken (10 ml), concentrated, and preserved. Mean numbers of larvae/ml including the standard error (±SE) were calculated for each water column sample of each pseudo-replicate set. Throughout the assay embryos of the water controls were checked for normal development and samples of key developmental stages were taken for RNA extractions.

### Gene expression

The effect of treatment on the gene expression of CgRXR, CgRAR and CgPPAR (not differentiated between isoforms) in trochophore and D-shelled larvae was tested by using quantitative RT-PCR (qPCR). One microliter of total RNA of treated larvae and controls (water & DMSO) were reverse transcribed and following qPCRs with gene specific primers and reference genes including additional statistics were performed as previously described [63]. No RNA could be extracted for ATRA high which was therefore excluded from this analysis.

## Results

### Isolation, phylogeny and ligand binding modelling of *C*. *gigas* nuclear receptors CgRXR, CgRAR and CgPPAR

**Fig 1:**
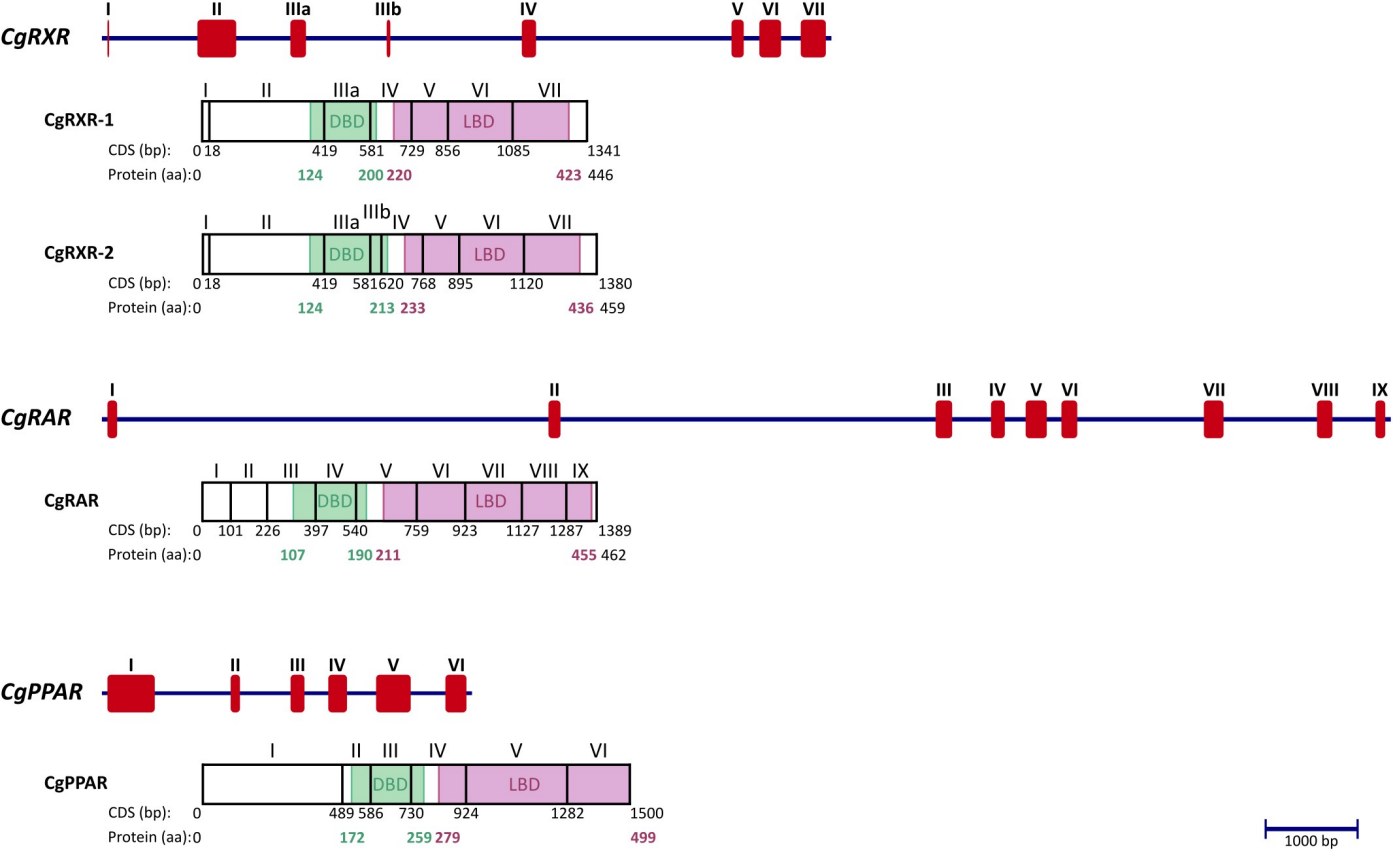
Exon/intron structure, coding sequence and protein organization of CgRXR-1, CgRXR-2, CgRAR and CgPPAR. Blue line: genomic sequence (bp); red rectangles: exons forming CDS (bp); roman numerals: number of exon; Arabic numbers: position and length for either CDS (bp) or protein (aa). Green boxes/numbers: DBD position in protein; purple boxes/numbers: LBD position in protein.

**Fig 2:**
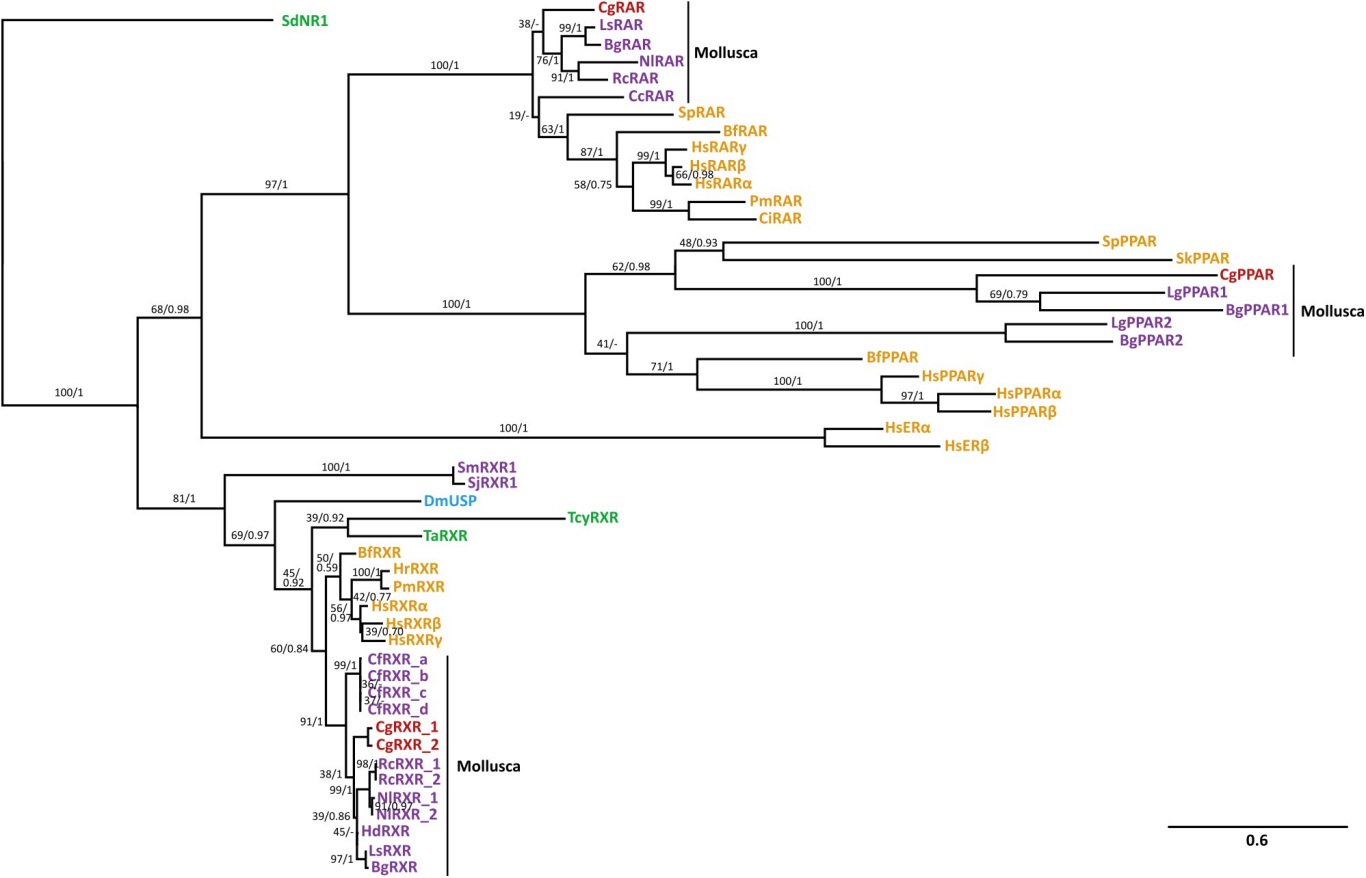
Phylogenetic tree of nuclear receptor homologs RXR, RAR and PPAR among various phyla. The alignment was constructed using the DBD plus portion of LBD and phylogenetic relationship was conducted by Maximum Likelihood and Bayesian Inference. Maximum Likelihood bootstrap support values (percentage of 1000 BS) and Bayesian posterior probabilities are provided above the nodes separated by slash. The *Suberitus domuncula* SdNR1 receptor was used as outgroup. Bf: *Branchiostoma florida*; Bg: *Biomphalaria glabrata*; Cc: *Ciona intestinalis*; Cf: *Chlamys farreri*; Cg: *Crassostrea gigas;* Dm: *Drosophila melanogaster*; Hd: *Haliotis diversicolor*; Hr: *Halocynthia roretzi*; Hs: *Homo sapiens*; Lg: *Lottia gigantea*; Ls: *Lymnaea stagnalis*; Nl: *Nucella lapillus*; Pm: *Polyandrocarpa misakiensis*; Sj: *Schistosoma japonicum*; Sk: *Saccoglossus kowalesvski*; Sm: *Schistosoma mansoni*; Sp: *Strongylocentrotus purpuratus*; Ta: *Trichoplax adhearens*; Rc: *Reishia clavigera*; Tcy: *Tripedalia cystophora*. ER: estrogen receptor. Red: *C*. *gigas* receptors; Orange: Deuterostomia; Purple: Lophotrochozoa; Blue: Ecdysozoa; Green: other metazoans.

The genomic DNA data of each receptor was used as template for sequencing the full CDS (coding sequence) of three oyster NRs and their isoforms. Alignment of CDS to the genomic DNA sequence showed clear exon/intro structure for each NR (Fig 1). Each CDS encodes for a protein which includes the expected domain structure of NRs: a highly variable A/B domain (N-terminal), a DBD, a flexible “hinge” region in between, a LBD, and for most receptors a final highly variable F-domain (C-terminal). Furthermore, two conserved zinc finger motifs of the DBD and additional features, which show high homology to those used for dimerization, cofactor recognition and ligand binding, were also identified (S1 Fig). Phylogenetic analyses (maximum likelihood and Bayesian Inference) of DBD plus LBD amino acid alignments were conducted to confirm homology for each NR (Fig 2). The sponge *Suberitus domuncula* SdNR1 was used as outgroup of the combined tree of both analyses. Sponge NR1 receptors are assumed as the one of two most ancient nuclear receptors [64]. The *in silico* 3D models for CgRXR and CgRAR were successfully created based on crystal structures of human homolog NRs bound to specific ligands. Computational docking calculated the binding affinity for retinoids (9RA, ATRA, TTNPB) and TBT to CgRXR and CgRAR models (Table 1, S2 Table). The same ligands, docked to human source models, provide reference affinity values for successful binding, possible induction of conformational change and signal transmission. Models of the 3D structure of CgPPAR LBD were considered unreliable as its sequence identity to human homologs is low (<24%) (S3 Table), but docking of TBT and rosiglitazone to these models was still conducted for comprehensiveness. Binding probabilities of CgPPAR to PFOA could not been tested as no human HsPPARα template bound to PFOA were available. Binding affinity values between positive and negative controls show small differences. Docking of 9RA to the positive control model of HsRXRα (pdb ID: 1FBY) resulted in binding affinities of -10.6 kcal/mol (S2 Table). The negative controls estradiol and ATRA, which do not induce agonistic signals in HsRXRα, were assigned less negative binding affinities (-9.5 & -9.1 kcal/mol, respectively), but displayed ligand positioning unlikely to induce conformational changes when docked to the human model. These comparably small differences between the docking energies of the negative and positive controls are a drawback of the used docking method. The docking of the expected natural ligand was usually accompanied by hydrogen bond/salt bridge formation and by a better fit of the hydrophobic part of the ligand into the receptor cavity. This results in lower energy values in comparison to the negative controls. The high binding energy values for the negative controls, on the other hand, are probably due to the binding of the bulky hydrophobic ligands into the corresponding LBD pockets, including a burial of the significant hydrophobic surface area.

**Table 1:** Calculated binding affinity values (kcal/mol) by computational docking of retinoid ligands to the created 3D models of human, oyster and gastropod retinoic acid receptors (RARs). (VGSMNL): single-letter code of residues changed in CgRAR ligand binding domain; pdb template: pdb ID providing the template for 3D modelling; RA: retinoic acid; ATRA: all-*trans* RA; Cg: *Crassostrea gigas*, Hs: *Homo sapiens*; Nl: *Nucella lapillus*; Rc: *Reishia clavigera*

Ligand	Receptors						pdb template ID
	HsRARα HsRARγ HsRARβ	CgRAR	RcRAR	NlRAR	CgRAR (VSGMNL)	NlRAR (mutated)	
9-*cis* RA	-11.7	-10.2	-9.8	-10.3	-11.8	-11.6	3LBD
ATRA	-11.8	-10.5	-9.7	-9.8	-11.4	-11.0	2LBD
TTNPB	-14.9	-12.6	-11.6	-12.2	-14.7	-14.7	1XAP

#### Retinoid X receptor CgRXR

Two isoforms for the oyster RXR homolog were identified and named CgRXR-1 and CgRXR-2, encoding a 446 amino acid (aa) and a 459 aa protein, respectively (GenBank accession numbers: CgRXR1: KX590999; CgRXR2: KX591000) (Fig 1). The difference between CgRXR-1 and CgRXR-2 is a 13 aa long insertion/deletion in the T-box of the DBD. Sequence alignment to known RXR homologs in other mollusc species shows that the T-box, a conserved section of the DBD required for dimerization, is likely to be common for molluscan RXR isoforms (S1 Fig). However, the CgRXR-2 sequence is unique and does not show any similarities to other molluscan isoforms, suggesting it is a Pacific oyster specific insertion. Sequence identities for the conserved regions to other molluscan RXRs range from 90–97% for the DBD and 90–93% for the LBD (S3 Table). Identical P-box sequences (first zinc finger; ‘CEGCKG’), a DNA recognition motif, were identified for human and molluscan RXRs. The D-box (second zinc finger; ‘RDDRN’), responsible for dimerization, shows 100% identity to the bivalve *C*. *farreri* and one amino acid difference to gastropod RXR’s D-boxes (‘R’). Conserved domain searches identified similarities to features known for DNA binding, ligand binding and co-activator recognition sites. A homodimer interface, recognised as a requirement for RXR homodimers on response elements DR1 and DR2 (two half site motifs as direct repeats (DR) separated with a short spacer of one or two nucleotides), was also identified, as well as equivalent heterodimer interfaces for RAR, PPAR and ecdysone receptor (ECR) heterodimer formations. The phylogenetic analysis places both CgRXR isoforms inside the group of other known molluscan RXRs (Fig 2). The high sequence identity of the LBD of CgRXR-1 to human RXRs allowed reliable CgRXR 3D models to be built. Computational docking showed similar binding affinities (-10.6 kcal/mol) of 9RA to CgRXR, using HsRXRα model as a positive control, and to the two snail receptors NlRXR and RcRXR (S2 Table), which are both known to respond to 9RA [16, 41]. The oyster RXR ligand docking position was comparable to human RXRα (S2 Fig). Binding affinities to TBT were also consistent between the CgRXR model, the HsRXRα control model (pbd ID: 3E94) and TBT responding RcRXR [16] models (-5.4 ˗ -5.6 kcal/mol) (S2 Table). According to these results, TBT does not bind tightly to the receptor. However, the modelling always positions the tin atom in the vicinity of the side chain of the conserved cysteine C415 in the H11 helix.

#### Retinoic acid receptor CgRAR

**Fig 3:**
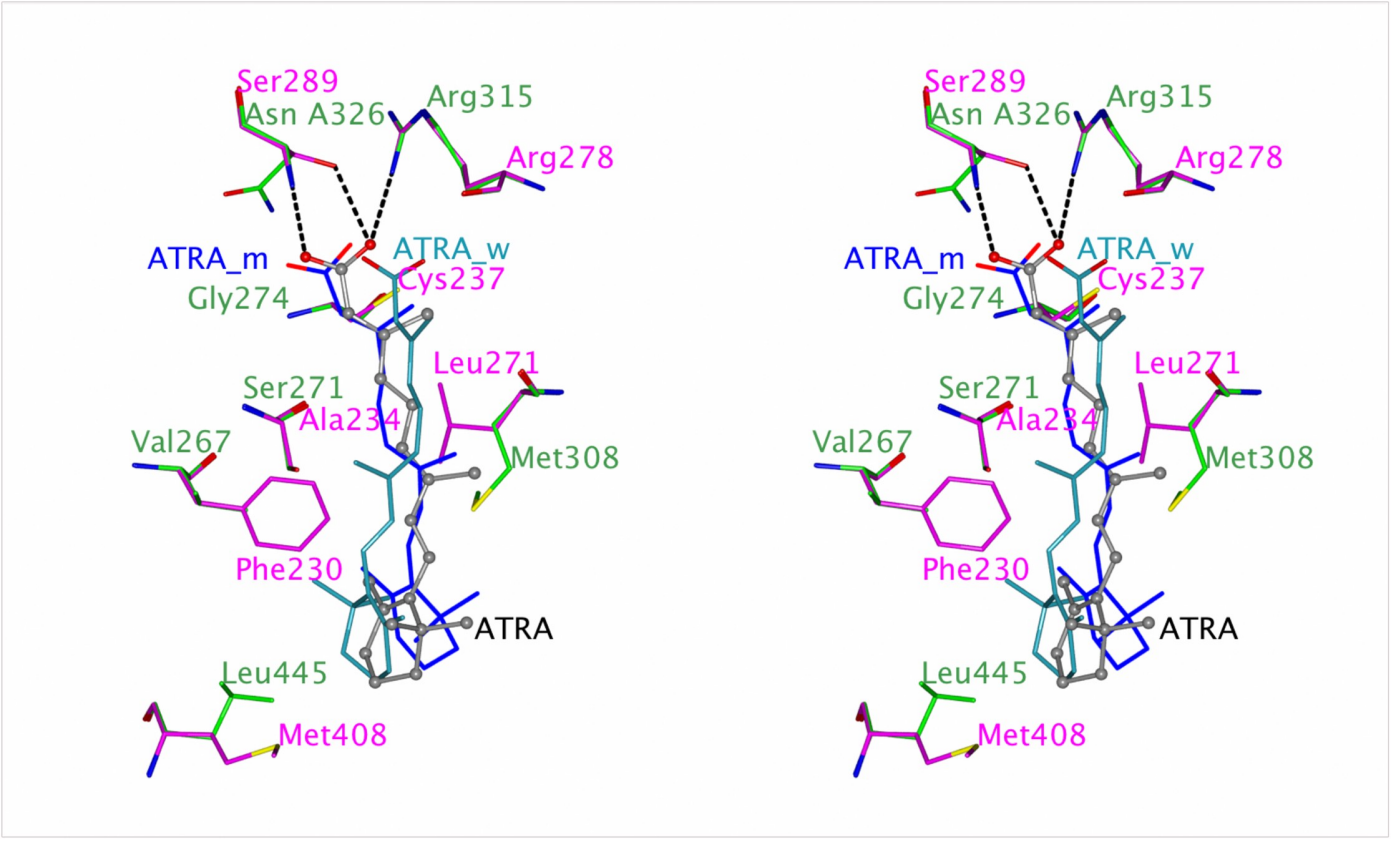
Stereo view of ATRA bound to the ligand binding pocket of a CgRAR model. Superimposition of model CgRAR (purple) on the crystal structure of HsRARγ LBD (green) bound to human RAR agonist ATRA. Original ATRA (grey) bound to HsRARγ LBD template (pdb ID: 2LBD); ATRA (light blue) to CgRAR wildtype, ATRA (dark blue) bound to mutated CgRAR. Divergent residues as well as arginines binding to the COOH group of ATRA including hydrogen bonds are indicated.

Nine exons form the 1389 base pair (bp) long CDS of CgRAR, which encodes a 462 aa long protein (GenBank accession number: CgRAR: KX591001) (Fig 1). Additional RAR isoforms could not be confirmed by three independent cloning attempts, but sequencing of one, possibly rare isoform, suggests presence of a CgRAR isoform showing two supplementary amino acids in the T-box of the DBD (S1 Fig). Sequence identity to three gastropod RARs ranged from 90–95% for the DBD and 58–60% for the LBD, respectively (S3 Fig). The P-box (‘CEGCKG’) is identical to its molluscan and human homologs. The D-box (‘HKDKN’) shows differences to human (‘HRDKN’), *L*. *stagnalis* (‘HKEKN’) and *N*. *lapillus* (‘HKDQT’), but it is identical to the D-box of *R*. *clavigera*. DNA, ligand and co-regulatory recognition sites as well as the heterodimer interface site could also be recognized. CgRAR groups together with related molluscan RARs (Fig 2). Computational docking suggests that CgRAR is unlikely to be activated by RAs. Neither 9RA, ATRA nor TTNPB occupy the correct position to cause required induced conformational changes in the receptor for signal transduction. Modelled CgRAR binding affinity energies are similar to RcRAR and NlRAR (Table 1), which were both shown not to respond to RAs *in vitro* [41, 42]. Human RARs, on the other hand, are able to respond to RAs and accordingly display better binding affinities (more negative energy values) to different types of RAs. When analysing the ligand binding of ATRA to CgRAR, six residues could be identified, which prohibit the binding required for the ‘induced fit’ conformational changes (Fig 3). Three residues (S271, M308, N326) sterically prohibit the correct ligand positioning, including its carboxyl group, which in turn results in a weakening of the salt bridge to arginine (A315). Three additional residues (V267, G274, L445) are less bulky than their equivalents in human RARs. They match the surface of the ligand unfavourably and cannot provide the required induced fit conformational change in the receptor domain. When these six residues in CgRAR were changed into the equivalent HsRARs residues, the ligand binding affinity of ATRA, 9RA and TTNPB to CgRAR reverts to the human RA affinities (Table 1). Ligand position and induced fit become more advantageous for an induction of conformational changes, suggesting a recovery of ligand response (Fig 3). Similar results have been reported for *N*. *lapillus* RAR [41]. Seven residues in the ligand binding pocket of NlRAR, of which five are shared with CgRAR, have previously been shown to prevent the receptor from responding to retinoic acid. All six changed residues result in a ligand binding affinity increase similar to the HsRAR level (Table 1).

#### Peroxisome proliferator-activated receptor CgPPAR

CgPPAR, the longest of the three investigated receptors, is a six exon, 1500 bp long sequence, encoding for a 499 aa long protein (GenBank accession number: CgPPAR: KX591002) (Fig 1). Sequence identity shows medium identities (75–78%) for the DBD to PPARs identified in other molluscan species, but a low sequence identity for the LBD (29–38%) (S3 Table). The LBD is also shorter than the human LBD (approx. 50 aa) and sequence alignment indicates an absence of the helixes H2, H2’ and H12 (S1 Fig). Although the P-box (‘CEGCK’) is identical, the D-box (‘ENPKG’) does not show any similarities to other molluscan or human PPARs. CgPPAR groups together with homologous PPARs, and gastropods BgPPAR1 and LgPPAR1 are the closest identified relatives to CgPPAR (Fig 2). An additional CgPPAR such as a homolog to the gastropod BgPPAR2 or LgPPAR2 could not be identified and PPAR2 homologs seem to be lost in *C*.*gigas*. The reliability of 3D structure models of CgPPAR are limited by the low sequence homology to the only physically characterised PPAR receptor: HsPPARγ, and hence computational docking results for this receptor are less trustworthy. Rosiglitazone is predicted to dock to the CgPPAR model with similar binding affinity values (~-8.2 kcal/mol) to HsPPARγ (pdb ID: 4EMA) (S2 Table), but its positioning does not suggest an induction of a conformational change. TBT docks loosely to the hydrophobic pockets of CgPPAR and HsPPARγ (pdb ID: 3WJ4) (-5.1 kcal/mol) and positions its tin atom towards a cysteine in the H3 helix in both receptor models.

### Embryo toxicity tests

**Fig 4:**
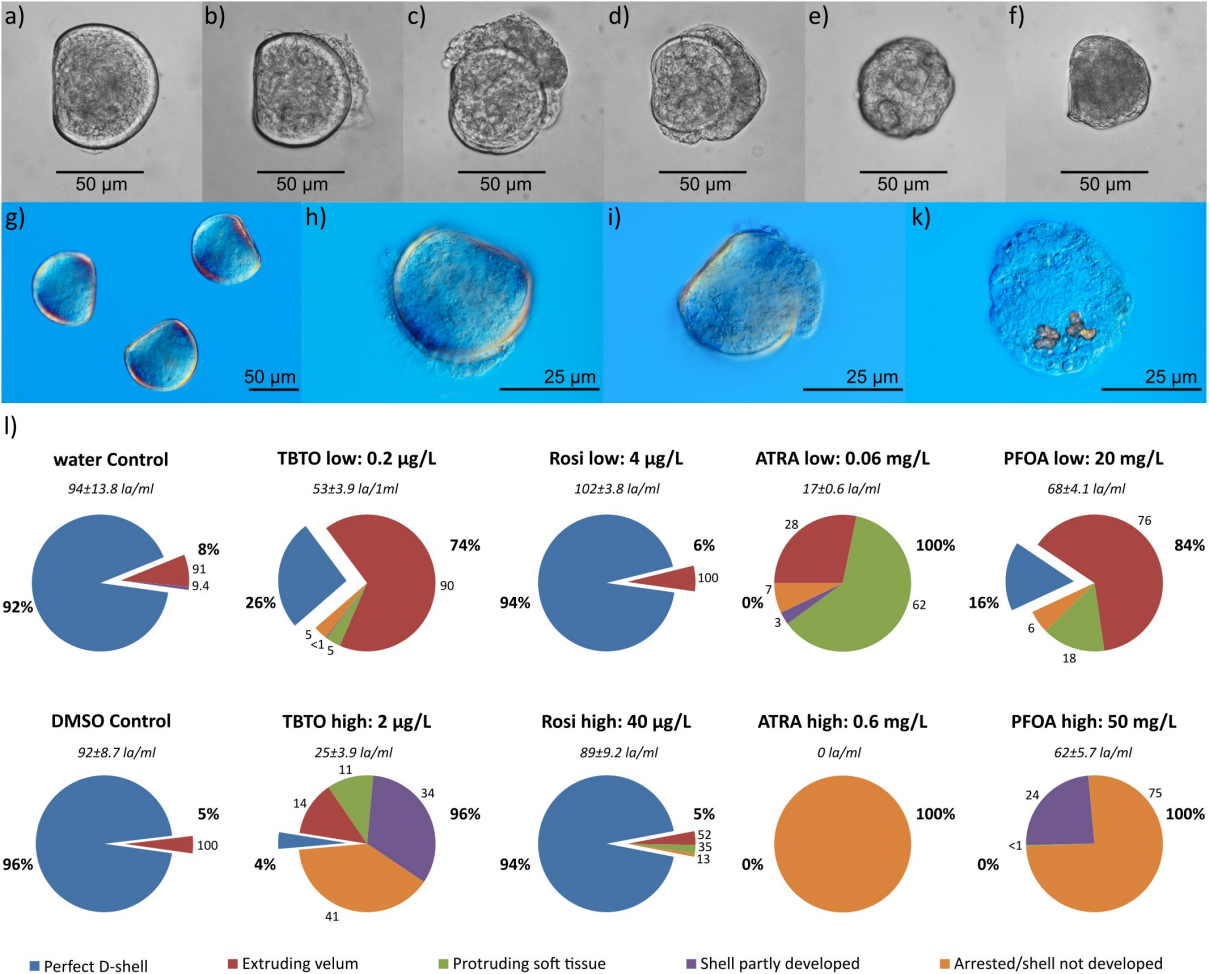
Oyster embryo development after 21 h of exposure to TBTO, rosiglitazone (Rosi). **All-*trans* retinoic acid (ATRA) and perfluorooctanoic acid (PFOA). a–k)** Example of oyster development under a light (grey) and differential interference contrast (blue) microscope. **l)** Percentage of perfect developed D-shaped larvae and abnormal developed larvae grouped in in four abnormal development categories. Bold numbers next to pie charts: percentage perfect D-shaped (left) and total abnormal D-shaped (right) larvae. Non-bold numbers: percentage of abnormal developed categories to total percentages of abnormal developed D-shaped larvae. The standard error of percentage larval development did not exceed ±6% (not shown). Italic numbers: swimming larvae in the water column per ml (la/ml). Oyster individual categories: perfectly developed D-shaped larvae (**a, g;** blue), extruding velum (**b, h;** red), protruding soft tissue, (**c, I;** green), shell partly developed (**d;** purple), arrested shell/shell not developed (**e-f, k;** orange).

The effects of TBTO, rosiglitazone, PFOA and ATRA on oyster development were tested by oyster embryo bioassay. Larval appearance and developmental status were microscopically assessed after completion of the assay (24 hpf) (Fig 4). Five categories of larval appearance could be identified. Development of a perfect D-shaped larva (Fig 4A and 4G) indicates a normal development of an oyster larva at around 24 hpf. The abnormal group includes four categories: (1) extruding velum, exposing structures of the velum such as the cilia (Fig 4B and 4H); (2) protruding soft tissue, showing enlarged soft tissue at one side of the normal sized D-shell (Fig 4C and 4I); (3) shell partly developed, development of a much smaller D-shaped shell with much the animal’s body exposed (Fig 4D); (4) arrested/ shell not developed, including individuals for which development is arrested at the trochophore larval stage (Fig 4F, 4E and 4K) or atrophied larval animals without a shell (Fig 4F). Arrested and no-shell development could not be clearly distinguished under a standard light microscope, and were therefore grouped together. This last category is rated as the most severe as it does not allow progression to D-shaped larval stage including shell development. Differential interference contrast microscopy showed that in the latter group of individuals had, on occasion, developed shell-endowment/disposition (Fig 4K, orange-red structure), but could not continue to develop a full D-shaped shell. The reference treatment with zinc showed an increasing negative effect on the development perfectly D-shaped oyster embryos with increasing zinc concentration (S3 Fig).

The untreated water controls show high percentages of perfectly developed D-shaped larvae (92%) (Fig 4L). The solvent control (DMSO) did not have any visible effects on the oyster development, and neither did the chemical rosiglitazone (perfect D-shaped larvae: 94–96%). TBTO, ATRA and PFOA, on the other hand, have severe effects on oyster development at both, low and high, concentrations. TBTO, the chemical with the lowest concentration (0.2 μg/L), displays effects on the oyster development resulting in only 26% normal perfectly developed D-shaped larvae. A tenfold increase in TBTO concentration (2 μg/L) leads to more severe effects with almost 40% of embryos not even reaching the D-shaped larval stage. This is comparable to previously reported data of oyster embryos exposed to TBT for 24 h with an LC_50_ of 3.9 μg/L [12] and an EC_50_ of 1.7 μg/L [65]. The effects of TBTO also intensified at lower embryo concentration (data not shown). At a ten times lower initial embryo concentration (20 embryos/ml) at low TBTO concentration 18% normal development occurred and at high concentration all embryos are arrested or the shell could not been developed. Low dose of ATRA (0.06 mg/L) appears to disturb organ and tissue development as well as shell formation. Most of the individuals exposed to a low dose of ATRA show normal sized shell development, but have extruding velum or protruding soft tissue. Exposure of the pond snail *L*. *stagnalis* to range of ATRA doses (10^−7^–10^−5^ M) displayed comparable levels of organ (eye) and shell deformations as well as arrested trochophores during early development [39, 40]. At the higher concentration of ATRA (0.6 mg/L) none of the oyster individuals reached the D-shaped larval stage. Microscopic examination of the high ATRA samples showed no surviving individuals, not even as arrested trochophore individuals. PFOA has a similar impact on development, albeit at much higher concentrations. The lower concentration (20 mg/L) leads to extruding velum, protruding soft tissues and enlargements. The higher concentration (50 mg/L) prevents embryos from developing a full size D-shaped shell or reaching the D-shaped larval stage. This concurs with measured effective concentrations of PFOA in microalgae, marine invertebrates and fish (EC_50_: 12–164 mg/L) [66].

The primary measures of effect from the oyster embryo bioassay are measured on the animals that remain in the water column (as free swimming or floating larvae) but in fact the effects of the chemicals can also been seen in the actual numbers of larvae that remain in the water column. While approximately half of the controls and rosiglitazone larvae were swimming in the water column TBTO, ATRA and PFOA showed a decrease in floating/swimming larvae (Fig 4).

The effect of chemicals on expression of three NR genes was tested with qPCR at trochophore larval and D-shaped larval stages (S4 Fig). The three receptors are expressed in both larval stages with CgRXR and CgRAR (water controls) having significantly (p<0.05) higher expression in trochophore larvae. The expression patterns and levels are comparable to previous reported [63]. TBTO, rosiglitazone, PFOA or ATRA do not to alter the expression of these receptor genes in any of these exposed oyster individuals.

## Discussion

### Cloning and phylogenetic analyses of *C*. *gigas* nuclear receptors

We cloned three NRs, namely CgRXR, CgRAR and CgPPAR. CgRXR shows two different isoforms, CgRXR-1 and CgRXR-2. CgRXR-2 has a 13 aa long insertion in the T-box, a locus typically seen for different isoforms of RXR homologues in molluscs. All receptors display the distinct NR domains including DBD and LBD for hypothetical/potential DNA and ligand binding, respectively. CgRXRs and CgRAR show high sequence identities for the conserved domains (>82% DBD; 50–94% LBD) to molluscan homologues and even to remotely related species such as the human RXRs. CgPPAR, on the other hand, displays much lower sequence identities to the two other identified molluscan and the three human PPARs (56–78% DBD; 22–38% LBD). Phylogeny of the receptors confirms this conservation pattern (Fig 2). Sequence analysis, identities and phylogeny are used to make assumptions for the *C*. *gigas* NR functions.

### DNA binding and dimerization potential

DNA binding, the most important mechanism for NR mediated regulation of gene transcription, is mainly achieved by the DBD binding to response elements in promoters of the genes to be transcribed [1]. In addition to general DNA binding sites, the P-box in the first zinc finger contains residues necessary for sequence discrimination of response elements. All three oyster receptors contain identical P-box sequences (‘CEGCKG’) to the NR orthologs in humans and gastropods, leading to the assumption of a similar binding behaviour to specific response elements in the oyster receptors. Human RXR, RAR and PPAR homologs recognise response elements with direct repeats (DR) of various length (5’-AGGTCA(nx)AGGTCA-3’) [67]. For the gastropod rock shell *R*. *clavigera* RcRXRs, freshwater snail *B*. *glabrata* BgRXR and dog whelk *N*. *lapillus* NlRXR-1, binding to DR1, DR2 and/or DR5 response elements has been confirmed [16, 41, 68]. Similar findings were shown for *N*. *lapillus* NlRAR [41] and *R*. *clavigera* RcRAR, for which a variant of the DR5 response element (5’-AGTTCA-3’) was used [42]. Human PPARs bind to DR1 and DR2 response elements [21, 69].

Vertebrate RXRs are known to form homodimers, but also for being the predominant heterodimer partner for other receptors such as RAR and PPAR [20, 70]. Dimerization is a complex process observed in many receptors. It involves several receptor domains such as the DBD and LBD including the D- and T-boxes, and the homo and heterodimer interfaces [20, 71, 72]. CgRXR-1 and CgRAR both show identical residues to their molluscan homologues for the RXR/RXR homodimer and RXR/RAR heterodimer interfaces as well as for T- boxes. In gastropods BgRXR, RcRXRs and NlRXR-1 homodimer formation and binding to the response element DR1 as well as heterodimerization of RcRXR-1/RAR (DR5) and NlRXR-1/RAR (DR1, DR2 & DR5) have been confirmed [16, 41, 68]. The high sequence identity and dimer interface identification suggests successful homodimerization of CgRXR-1, and that heterodimerization of CgRXR-1 and CgRAR is possible. The CgRXR-2, however, might not be effective in homodimer formation. The isoform insertion is localised in the T-box, which might inhibit the dimerization as indicated for RcRXR-2 [16]. Few differences were detected between the RAR D-boxes of the different mollusc species, which could indicate that heterodimerization is not possible. However, the D-boxes, normally involved in dimerization of many heterodimers [20, 72], seem not to be necessary for all heterodimer formations. Human RXR/RAR heterodimer binding to the DR2 response element exclude the D-box in their dimerization process [71]. Human PPARs are strictly heterodimers and only bind to RXRs, which dimerise via the T-box only, excluding the D-box from the process [73]. Hence, the differences in length and sequence of the D-box for CgPPAR and human PPARs, as shown by our alignment results (S1 Fig), would not prevent heterodimerization of the oyster PPAR. Sequence analysis reveals a putative dimer interface in CgPPAR, and CgRXRs show RXR/PPAR heterodimer interfaces.

### Ligand binding potential

The ability to bind ligands is a common feature of RXRs, RARs and PPARs in many species [1, 20]. All three *C*. *gigas* receptors possess a LBD, including ligand binding sites. Sequence consensus between oyster, molluscan and human RXRs are very high for the LBD. The residues with which ligands bind are identical. Gastropod and human RXRs have been shown to bind to 9RA and TBT [16, 41, 42, 68]. The *in silico* 3D models of CgRXR and computational docking show a high likelihood that 9RA and TBT induce conformational changes in CgRXR, which would cause a ligand-dependent effect. Similar ligand binding positioning in human and oyster models are seen for 9RA including an induced fit in the hydrophobic pocket. This suggests binding of 9RA to CgRXR and a possible induction of an agonistic signal equivalent to human or snail RXRs. In HsRXRα the tin atom of the TBT molecule covalently binds to a cysteine thiol in the H11 helix [18] and induces agonistic conformational changes for receptor dimer activation. In oysters this cysteine (C415) is conserved (S1 Fig) and with TBT preferred binding exposing its tin atom to the cysteine, we hypothesise that CgRXR is potentially able to respond to TBT.

In contrast to human RARs, which are able to bind a variety of natural and synthetic retinoic acids such as 9RA, 13RA, ATRA and synthetic agonists (e.g. TTPNB) [31, 43], CgRAR may be unable to respond to such ligands. Based on the 3D models and the computational dockings, six residues in the LBD of the oyster RAR prevent binding of retinoids in the required conformation. Taking into account that neither of the two gastropod receptors, RcRAR or NlRAR, respond to RAs *in vitro* [41, 42], it is possible that molluscan species in general do not respond to RAs. This supports the theory of ligand binding loss for molluscan RARs [41]. It has been proposed that the urbilaterians, the last common ancestor of bilaterians before they split into deuterostomes and protostomes, possessed a proto-RAR, which was able to respond to RAs. Accordingly, loss of ligand binding could have emerged through just a few amino acid mutations in the LBD. In the dog whelk *N*. *lapillus*, ligand binding of NlRAR to 9RA and ATRA could be artificially restored *in vitro* through single or multiple mutations of up to seven amino acids to the equivalent human residues. CgRAR and NlRAR share five of the residues, known to prevent successful binding. Both receptors, when these residues are changed to the equivalent in human homologs, display binding energies similar to human retinoid binding RARs. CgRAR seems to have lost its ability to respond to RAs due to similar changes in the LBD as seen for NlRAR.

CgPPAR is missing a human PPAR typical helical region, H2, which results in a smaller ligand binding pocket (LBP). The LBP in the human protein is considerably large (~1300 Å^3^), but ligands usually only occupy about 30–40% of the cavity [74]. Hence, the shorter LBD of CgPPAR does not necessarily make the LBP too small for successful ligand binding. Although the 3D model of CgPPAR is not reliable due to high discrepancy to the human reference model, we would like to highlight few results from *in-silico* binding for future research on oyster PPARs. Computational docking shows that agonistic ligands such as rosiglitazone and TBT still fit in the LBP of the CgPPAR models. Rosiglitazone was chosen as putative CgPPAR ligand as it is an antidiabetic drug designed to interact with the human ortholog PPARγ [44]. However, rosiglitazone does not seem to position itself correctly to induce conformational changes. TBT, on the other hand, could stimulate a signal. TBT binds to a cysteine C285 in the H3 helix of HsPPARγ with an ionic bond and acts as a weak agonist [18, 22]. In the generated CgPPAR model, a cysteine C322 in the H3 helix would be in the position to bind to the tin compound. In contrast, since CgPPAR lacks the final H12 including an AF-2, which is required for ligand-dependent activation in most NRs, CgPPAR may not be activated by ligand binding, or recruit different means of passing on the induction signal.

### Chemical effects on oyster embryos

The oyster embryo bioassay showed that chemicals, such as the natural compound ATRA and synthetic compounds like TBTO and PFOA, affect the oyster embryo development at different concentrations. These effects include visible impacts on shell development, as well as malformation of the soft tissue of the animal itself. The highest chemical concentrations lead to arrested development at the trochophore larvae stage and in few cases even to high mortality. TBTO affected embryo development at a low dose of 0.2 μg/L, comparable to previous research, which also reported extruding velum in *C*. *gigas* embryos after 24 h exposure to the lowest tested TBTO concentration (1 μg/L) and a LC_10_ of 0.36 μg/L [12]. We hypothesise that the observed effects of TBTO on oyster development are caused by disruption of the CgRXR function. Our computational docking results support an interaction of TBT and the oyster CgRXR receptor. Previous research also strongly suggests a correlation of observed effect in gastropods and RXR interaction with TBT [14, 15]. In seawater TBTO (Bu_3_-Sn-O-Sn-Bu_3_) breaks down into two TBT units (2Bu_3_-Sn^+^) [75], which are available to interact with RXRs. The permissive RXR/PPAR heterodimer has been previously suggested as the specific target for TBT. Expression profiles of TBT-exposed dog whelk *N*. *lapillus* showed alteration of genes potentially involved in PPAR signalling pathways [76]. TBT can activate the human RXR/PPAR heterodimer via RXR alone or possible by both partners [17, 18, 22, 23]. The putative interaction of TBT and CgPPAR indicated by our sequence and docking analysis supports this theory. CgRXR is expressed in trochophore larval stage, where shell forming is initiated and our previous research also showed that CgRXR and CgPPAR is highly present during an earlier larval stage (gastrula stage), when organs are developed [63]. Hence, a putative disruption effect of TBT on RXR or a RXR/PPAR heterodimer may occur during these developmental stages, which could have led to the observed effects on shell formation and soft tissue stages. *C*. *gigas* embryos, on the other hand, were not visibly affected by rosiglitazone. This supports our computational docking result, which indicates that rosiglitazone does not interact with CgPPAR. However, previous research on the gastropod *N*. *lapillus* suggested a RXR/PPAR heterodimer involvement in imposex formation after exposure to rosiglitazone, displaying similar effects to that of TBT-induced imposex [76]. Oysters and snail PPARs, even though closely related, may react differently to rosiglitazone.

The effects of ATRA on oyster development, on the other hand, are possibly not caused via a traditional agonistic relationship of ligand and retinoid receptors similar to vertebrates. Our computational docking results refute an agonistic vertebrate-like interaction between RAs and CgRAR. Studied gastropod RARs also showed no receptor activation by ATRA [41, 42].

Additionally, gene expression of CgRAR and CgRXR in ATRA-exposed D-shelled larvae did not change compared to untreated oyster larvae. Human orthologs of RAR are self-regulated via an agonistic response to ATRA [77, 78], which also affects the expression of RXR isoforms [79]. Oysters CgRAR and CgRXR do not vary in their expression suggesting a different regulation mechanism in *C*. *gigas* not related to ATRA. An indirect effect of ATRA on CgRXR is a potential explanation, based on suggestions of an isomerization process of RAs; ATRA would be converted to 9RA and 13RA, with 9RA consequently interacting with CgRXR. Indeed, this mechanism is thought to be present in several gastropod species [80].

Our data of PFOA, a perfluoralkyl carboxylate used as synthetic salt, confirms the minor risk by a direct exposure from PFOA as previously reported for marine species [66] and it displays toxicity at what would be high levels of milligrams per litre. Although the environmental concentrations of PFOA are low (oceanic/coastal waters: 15 pg/L– 190 ng/L) [81], this chemical has been classified as a substance of a very high concern due to its high persistence (non-degradable) and ubiquitous existence in terrestrial and aquatic habitats, atmosphere, food, drinking water, plants, animals including humans [66, 81–84]. The concern regarding its persistence and bioaccumulation abilities raises questions to its mode of action. PFOA is a known agonist of vertebrate PPARα and PPARβ [45] and successfully disrupts the PPAR pathways [85, 86]. Due to the lack of a PPAR template bound to PFOA and the inability to generate a reliable CgPPAR 3D model the possible interaction of PFOA with CgPPAR was not assessed. Nevertheless, the presence of a PPAR homolog in the Pacific oyster forms a starting point for further investigations of PFOA mode of action in protostome invertebrates.

## Conclusion

The modes of action of many disruptive chemicals such as TBT, ATRA and PFOA in invertebrates are far from being fully comprehended. Our study demonstrates the vulnerability of oyster larvae to disruption when exposed to these ligands, which illustrates the potential risks for marine invertebrates in certain polluted environments. Three nuclear receptors of the Pacific oyster were cloned and shown to potentially offer pathways through which chemicals may execute their disruptive function. Our *in-silico* binding computational analysis provides indications for binding capabilities of these receptors to said chemicals and can serve as a foundation for further investigation (e.g. *in-vitro* ligand-binding assays), which could verify these theoretical findings.

## Supporting information

S1 Table Primers for sequencing full length RNA sequences of CgRXR, CgRAR and CgPPAR. (PDF)

S2 Table Calculated binding affinity values (kcal/mol) by computational docking of retinoid ligands to the created 3D models of human, oyster and gastropod RXR and PPAR homologs. pdb template: pdb ID providing the template for 3D modelling; RA: retinoic acid; ATRA: all-*trans* RA; TBT: tributyltin; Cg: *Crassostrea gigas*, Hs: *Homo sapiens*; Nl: *Nucella lapillus*; Rc: *Reishia clavigera*
(PDF)

S3 Table Sequence identity (percentage %) of amino acid sequences of *Crassostrea gigas* CgRXR-1, CgRXR-2, CgRAR and CgPPAR to molluscan and human receptor homologs. Cg: *C*. *gigas*; Rc: *Reishia clavigera;* Nl: *Nucella lapillus*; Cf: *Chlamys farreri*; Bg: *Biomphalaria glabrata*; Lg: *Lottia gigantea*; Ls: *Lymnaea stagnalis*; Hs: *Homo sapiens*.
(PDF)

S1 File Full protein sequences of CgRXR-1, CgRXR-2, CgRAR and CgPPAR. (FA)

S2 File Amino acid alignment of DBD and LBD of nuclear receptor homologs RXR, RAR and PPAR across various phyla. Name code: species code + receptor homolog _ genebank ID.
(FST)

S1 Fig Sequence alignment of CgRXR, CgRAR and CgPPAR and their isoforms to molluscan and human homologs including sequence features and motifs. A/B domain partial; H1-12 (yellow): α-helixes of LBD; H2, H2’, H12 (orange): non-CgPPAR specific helixes; red: P-box; green: D-box; purple: T-box; grey: TBT interacting cysteine; blue: CgRAR & NlRAR shared residues; dark green: CgRAR specific residues; violet: NlRAR specific residues.
(PDF)

S2 Fig Stereo view of 9RA bound to the ligand binding pocket of a CgRXR model. Superimposition of model CgRXR (purple) on the crystal structure of HsRXRα LBD (blue) bound to human RXR agonist 9RA. Original 9RA (green) bound to HsRXRα LBD template (pdb ID: 1FBY); 9RA (pink) to CgRXR. Divergent residues as well as arginines binding to the COOH group of 9RA are shown as stick models. Hydrogen bonds are indicated as dashed lines.
(PDF)

S3 Fig Oyster embryo development after exposure to zinc and TBTO. Control exposure (200 embryos/mL) to increasing zinc concentration and water and DMSO Control, and TBTO low and TBTO high. Percentage of perfect developed D-shaped larvae (blue), and abnormal developed larvae grouped in four categories: extruding velum (**red**), protruding soft tissue, (**green**), shell partly developed (**purple**), and arrested shell/shell not developed (**orange**). Bold numbers next to pie charts: percentage perfect D-shaped (left) and total abnormal D-shaped (right) larvae. Non-bold numbers: percentage of abnormal developed categories to total percentages of abnormal developed D-shell larvae. The standard error of percentage larval development did not exceed ±6% (not shown).
(PDF)

S4 Fig Relative gene expression of CgRXR, CgRAR and CgPPAR nuclear receptors of oyster trochophore and D-shaped larvae exposed to TBTO, Rosi, PFOA and ATRA. Gene expression was measured with quantitative RT-PCR. Relative gene expression was calculated using a normalisation factor computed with the three reference genes and statistically analysed as described previously [63] and in the methods section. Bars indicate the mean ± standard error of three independent measurements per time point. Letters above each bar represent groups that were significantly different (p<0.05). * above water control samples show significant different expression between trochophore and D-shaped larval stage (p<0.05). wC: water Control; dC: DMSO Control; TL: TBTO low (0.2 μg/L); TH: TBTO high (2 μg/L); RL: rosiglitazone low (4 μg/L); RH: rosiglitazone high (40 μg/L); PL: PFOA low (20 mg/L); PH: PFOA high (50 mg/L); AL: ATRA low (0.06 mg/L).
(PDF)
