# Supplementary material for: Cloning retinoid and peroxisome proliferator-activated nuclear receptors of the Pacific oyster and in silico binding to environmental chemicals
Source: PLoS One. 2017 Apr 20;12(4):e0176024. doi: 10.1371/journal.pone.0176024 (PMC5398557; doi:10.1371/journal.pone.0176024)
Supplement: S1 Table — (PDF) [file pone.0176024.s001.pdf]

S1 Table. Primers for sequencing full length RNA sequences of CgRXR, CgRAR and CgPPAR.

Primer	Sequence
CgRXR_Fwd:	5’- ACAAAGGCATGGACCCATCAGA -3’
CgRXR_Rev:	5’- TCAAGTGCTGCTTGGGGATG -3’
CgRAR_Fwd:	5’- TGGGTGGTGTATGAAGACGGACA -3’
CgRAR_Rev:	5’- AGGAATACACACATTCTCCTGC -3’
CgPPAR_Fwd:	5’- AAGCACAATGGTGCACGGGT -3’
CgPPAR_Rev:	5’- CCATTGTCAGCAGTTGGTGTCATCT -3’
