# Supplementary material for: Cloning retinoid and peroxisome proliferator-activated nuclear receptors of the Pacific oyster and in silico binding to environmental chemicals
Source: PLoS One. 2017 Apr 20;12(4):e0176024. doi: 10.1371/journal.pone.0176024 (PMC5398557; doi:10.1371/journal.pone.0176024)
Supplement: S2 Table — pdb template: pdb ID providing the template for 3D modelling; RA: retinoic acid; ATRA: all-trans RA; TBT: tributyltin; Cg: Crassostrea gigas, Hs: Homo sapiens; Nl: Nucella lapillus; Rc: Reishia clavigera (PDF) [file pone.0176024.s002.pdf]

S2 Table. Calculated binding affinity values (kcal/mol) by computational docking of ligands to the created 3D models of human, oyster and gastropod retinoid X receptor (RXR) and peroxisome proliferator-activated receptor (PPAR). pdb template: pdb ID providing the template for 3D modelling; RA: retinoic acid; ATRA: all-*trans* RA; TBT: tributyltin; Cg: *Crassostrea gigas*, Hs: *Homo sapiens*; Nl: *Nucella lapillus*; Tc: *Thais clavigera*

Ligand	Receptors						pdb template ID
	**HsRXRα**	**CgRXR**	**TcRXR**	**NlRXR**	**HsPPARγ**	**CgPPAR**	
9-*cis* RA	-10.6	-10.6	-10.6	-10.5			1FBY
TBT	-5.6	-5.4	-5.5	-5.4			3E94
ATRA	-9.2	-9.1					1FBY
estradiol	-9.5	-9.6					1FBY
rosiglitazone					-8.4	-8.2	4EMA
TBT					-5.2	-5.2	3WJA
