# Supplementary material for: Cloning retinoid and peroxisome proliferator-activated nuclear receptors of the Pacific oyster and in silico binding to environmental chemicals
Source: PLoS One. 2017 Apr 20;12(4):e0176024. doi: 10.1371/journal.pone.0176024 (PMC5398557; doi:10.1371/journal.pone.0176024)
Supplement: S3 Table — Cg: C. gigas; Rc: Reishia clavigera; Nl: Nucella lapillus; Cf: Chlamys farreri; Bg: Biomphalaria glabrata; Lg: Lottia gigantea; Ls: Lymnaea stagnalis; Hs: Homo sapiens. (PDF) [file pone.0176024.s003.pdf]

S3 Table. Sequence identity (percentage %) of amino acid sequences of *Crassostrea gigas* CgRXR-1, CgRXR-2, CgRAR and CgPPAR to molluscan and human receptor homologs. Cg: *C. gigas*; Tc: *Thais clavigera;* Nl: *Nucella lapillus*; Cf: *Chlamys farreri*; Bg: *Biomphalaria glabrata*; Lg: *Lottia gigantea*; Ls: *Lymnaea stagnalis*; Hs: *Homo sapiens*.

NR	A/B	C*	T-box isoform	T-box length	D	E	F	Length (aa)
CgRXR-1								446
CgRXR-2	100 (1aa)	99 (1aa)	13 aa		100	98 (5aa)	100	459
TcRXR	60	94-95	-1:<br>-2:	-<br>5 aa	89	90-91	79 %	442<br>447
NlRXR	61	90-94	-1:<br>-2:	-<br>5 aa	89	90-92	75 %	441<br>446
CfRXR	69	96-97	-a:<br>-b:<br>-c:<br>-d:	-<br>4 aa<br>20 aa<br>24 aa	95	90-92	75 %	446<br>450<br>466<br>470
BgRXR	61-62	95-96	-		84	91	71	436
LsRXR	60	95-96	-		84	92-93	71	436
HsRXRα	46	88-90	-		63	81-83	67	462
HsRXRβ	12	82-83	-		37	77-78	67	533
HsRXRγ	41	86-87	-		32	80-81	67	463
CgRAR								462
TcRAR	14	95	-		46	60	85	472
LsRAR	18	91	-		64	58	93	478
NlRAR	14	90	-		25	60	97	365
HsRARα	20	86	-		39	51	2	462
HsRARβ	19	87	-		39	51	17	455
HsRARγ	25	90	-		43	49	24	454
CgPPAR								499
BgPPAR1	26	78			59	29		391
LgPPAR1	29	75			76	38		365
HsPPARα	14	56	-		14	22	-	468
HsPPARβ	9	56	-		14	23	-	441
HsPPARγ	13	57	-		0	24	-	505

* C domain (DBD) without isoform region in T-box
