# Supplementary figures and images for: Cloning retinoid and peroxisome proliferator-activated nuclear receptors of the Pacific oyster and in silico binding to environmental chemicals
Source: PLoS One. 2017 Apr 20;12(4):e0176024. doi: 10.1371/journal.pone.0176024 (PMC5398557; doi:10.1371/journal.pone.0176024)

[illegible]

b) Retinoid X receptor CgRXR

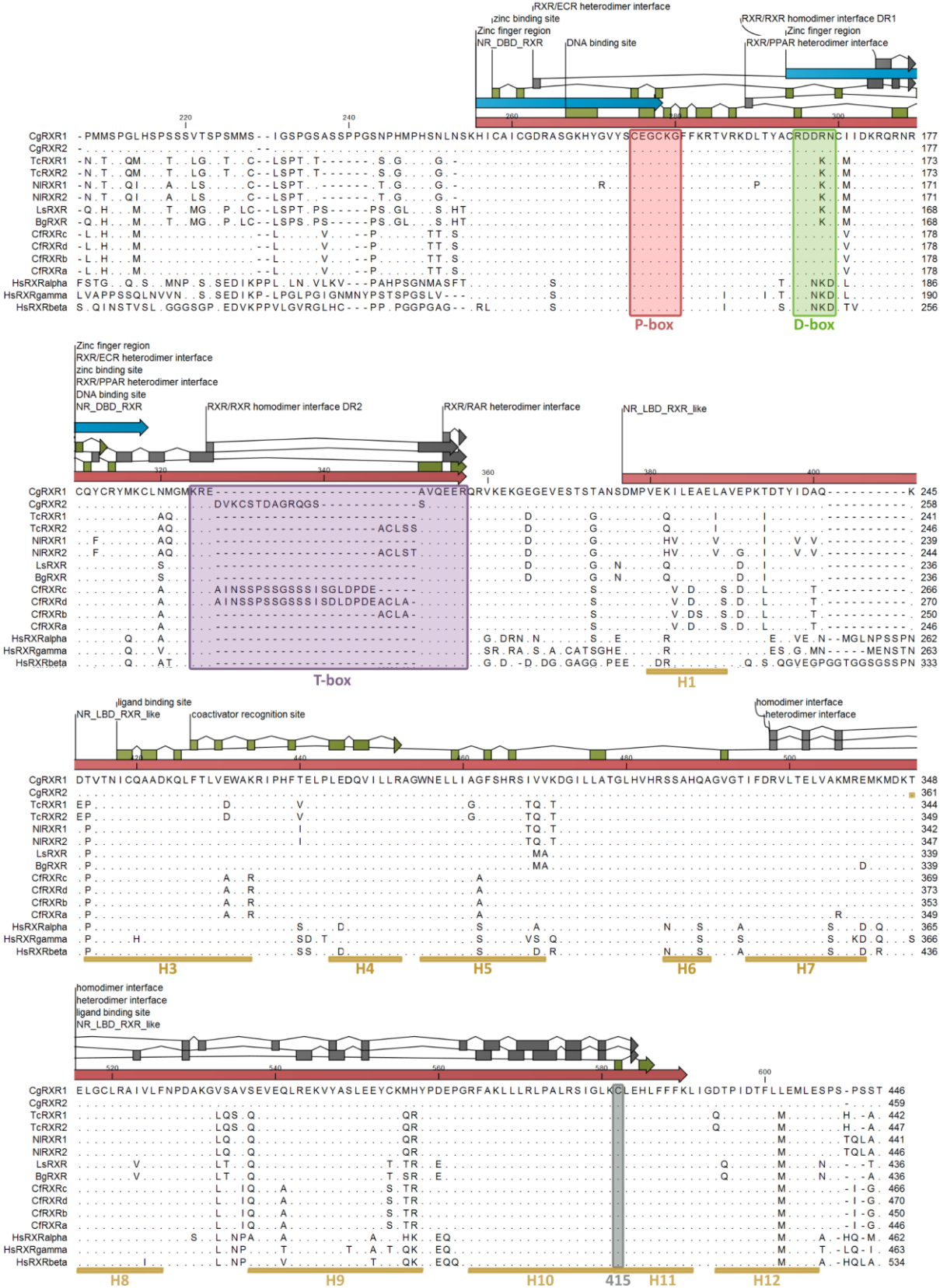

c) Peroxisome proliferator-activated receptor CgPPAR

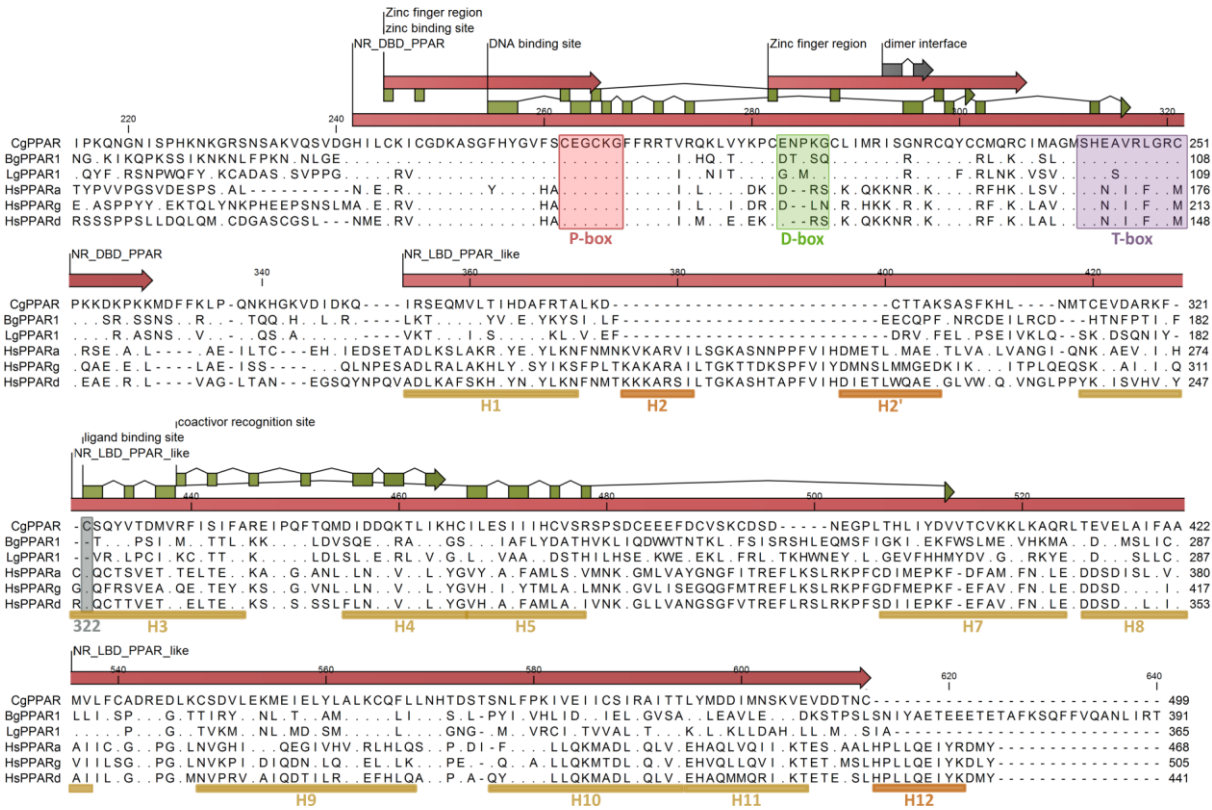

Supplement: S1 Fig — A/B domain partial; H1-12 (yellow): α-helixes of LBD; H2, H2’, H12 (orange): non-CgPPAR specific helixes; red: P-box; green: D-box; purple: T-box; grey: TBT interacting cysteine; blue: CgRAR & NlRAR shared residues; dark green: CgRAR specific residues; violet: NlRAR specific residues. (PDF) [file pone.0176024.s006.pdf]

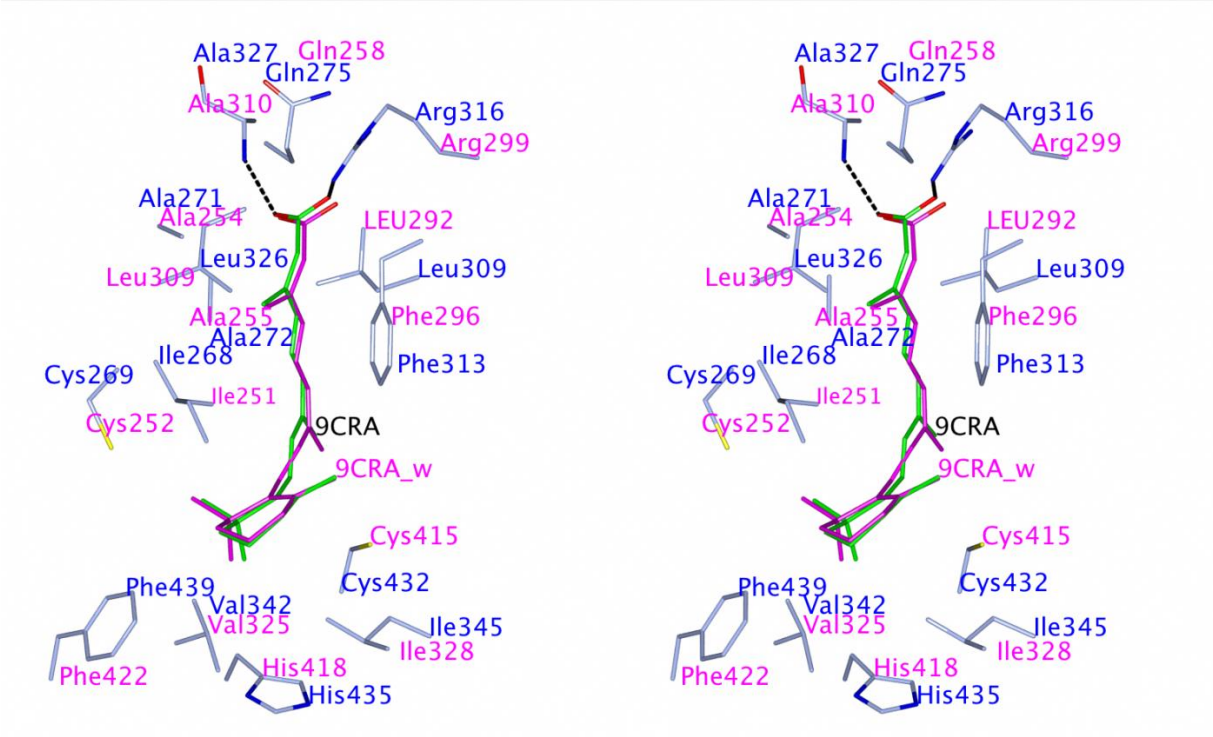

Supplement: S2 Fig — Superimposition of model CgRXR (purple) on the crystal structure of HsRXRα LBD (blue) bound to human RXR agonist 9RA. Original 9RA (green) bound to HsRXRα LBD template (pdb ID: 1FBY); 9RA (pink) to CgRXR. Divergent residues as well as arginines binding to the COOH group of 9RA are shown as stick models. Hydrogen bonds are indicated as dashed lines. (PDF) [file pone.0176024.s007.pdf]

## 200 embryos/mL

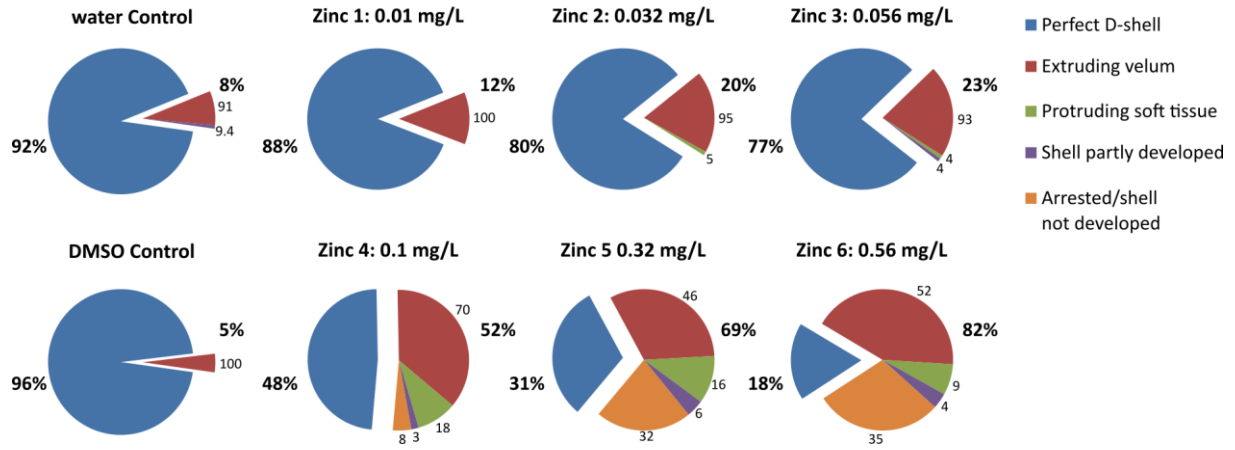

Supplement: S3 Fig — Control exposure (200 embryos/mL) to increasing zinc concentration and water and DMSO Control, and TBTO low and TBTO high. Percentage of perfect developed D-shaped larvae (blue), and abnormal developed larvae grouped in four categories: extruding velum (red), protruding soft tissue, (green), shell partly developed (purple), and arrested shell/shell not developed (orange). Bold numbers next to pie charts: percentage perfect D-shaped (left) and total abnormal D-shaped (right) larvae. Non-bold numbers: percentage of abnormal developed categories to total percentages of abnormal developed D-shell larvae. The standard error of percentage larval development did not exceed ±6% (not shown). (PDF) [file pone.0176024.s008.pdf]

## Trochophore larvae

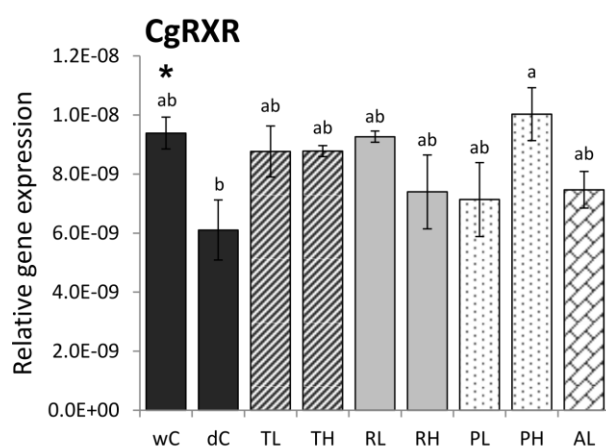

## D-shaped larvae

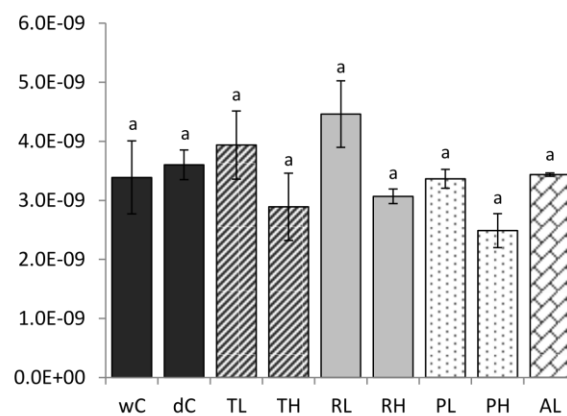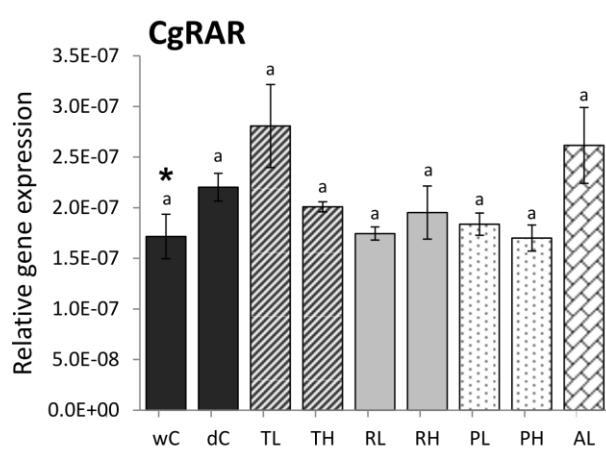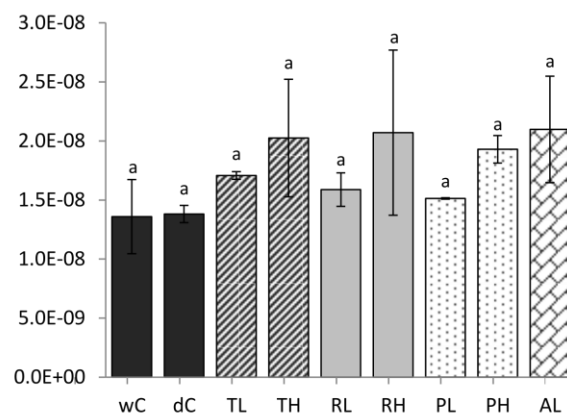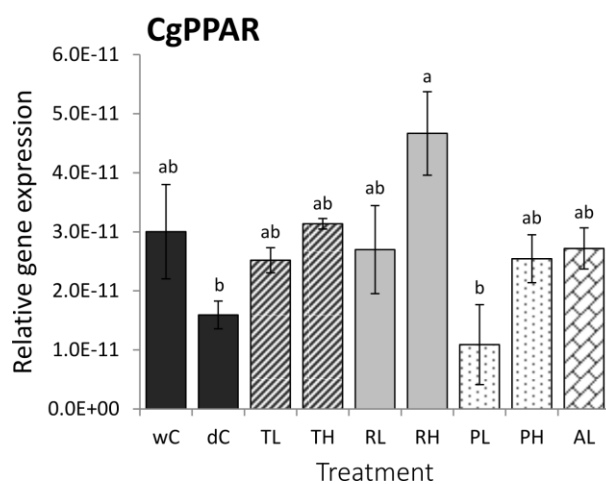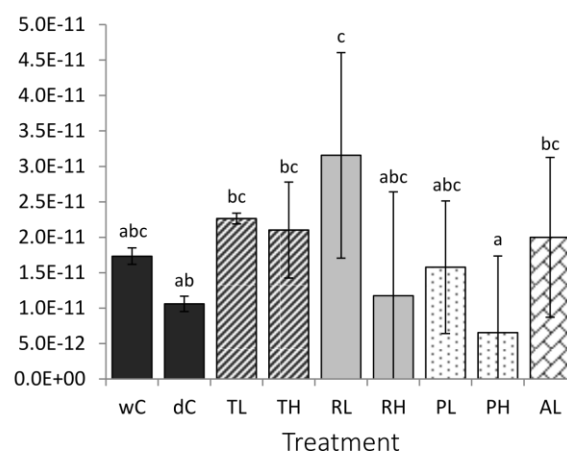

Supplement: S4 Fig — Gene expression was measured with quantitative RT-PCR. Relative gene expression was calculated using a normalisation factor computed with the three reference genes and statistically analysed as described previously [63] and in the methods section. Bars indicate the mean ± standard error of three independent measurements per time point. Letters above each bar represent groups that were significantly different (p<0.05). * above water control samples show significant different expression between trochophore and D-shaped larval stage (p<0.05). wC: water Control; dC: DMSO Control; TL: TBTO low (0.2 μg/L); TH: TBTO high (2 μg/L); RL: rosiglitazone low (4 μg/L); RH: rosiglitazone high (40 μg/L); PL: PFOA low (20 mg/L); PH: PFOA high (50 mg/L); AL: ATRA low (0.06 mg/L). (PDF) [file pone.0176024.s009.pdf]
